# Childhood Socioeconomic Status, Adult Personality, and Cognition: Early Indicators of Cognitive Impairment in Old Age

**DOI:** 10.1093/geroni/igaa057.1244

**Published:** 2020-12-16

**Authors:** Amanda Sesker, Jason Strickhouser, Páraic Ó Súilleabháin, Antonio Terracciano, Angelina Sutin

**Affiliations:** 1 Florida State University College of Medicine, Tallahassee, Florida, United States; 2 University of Limerick, Limerick, Ireland

## Abstract

This study investigated the association between childhood socioeconomic status (cSES) risk of cognitive impairment but not dementia (CIND), cognitive impairment (dementia or CIND), and dementia and whether adult personality mediated this association. A sample of 10,289 participants (aged 50 and older) from the Health and Retirement Study (HRS) were followed across 2-year periods between 2006 - 2018. Estimates of mediation effects in Cox Proportional Hazards regressions were conducted using Mplus software to approximate the total effects of cSES on the cognitive outcomes and the natural indirect effects and natural direct effects derived when personality causally mediated this outcome. cSES was associated with increased risk of all three cognitive outcomes. Conscientiousness partially mediated the relationship between cSES and dementia, CIND, and cognitive impairment risk while neuroticism partially mediated dementia and impairment, but not CIND. Personality improved the overall model fit between cSES and both CIND and impairment, and conscientiousness was specifically associated with significantly lowered cognitive impairment risk over time. Conscientiousness and neuroticism substantially mediated the relationship between cSES and risk of impairment in old age. This research adds to lifespan models and suggests that distinct personality traits attenuate early childhood factors that contribute to lifespan development and cognitive aging. Conscientiousness in particular may act as a protective buffer mediating risk factors associated with cognitive impairment in old age.

